# Rare case of an exceedingly enlarged mediastinal mass in 22-year-old male

**DOI:** 10.1093/jscr/rjac389

**Published:** 2022-10-30

**Authors:** Zamaan Hooda, Camillia Malhis, Luis Cerda, Mark Connolly

**Affiliations:** Department of Surgery, St. Joseph’s University Medical Center, Paterson, NJ, USA; Department of Surgery, St. Joseph’s University Medical Center, Paterson, NJ, USA; Department of Surgery, St. Joseph’s University Medical Center, Paterson, NJ, USA; Department of Surgery, St. Joseph’s University Medical Center, Paterson, NJ, USA

## Abstract

Masses of the mediastinum develop from anatomic structures that pass through or are normally located within this space; they may also occur secondary to metastatic spread from malignancies from other locations. The anterior compartment of the mediastinum can give rise to thymomas, which tend to be restricted to the thymus as well as nearby structures. Their symptoms are associated with their size and impact on nearby structures. This patient, a 22-year-old male with no medical history, presented to the emergency department for evaluation of a cough ongoing for 3 weeks. Chest X-ray and computed tomography (CT) scan of the thorax demonstrated a mass measuring up to 17.2 cm. Afterwards, a CT-guided biopsy was performed, which revealed findings consistent with a benign thymic neoplasm, though due to the specimen being scant, patient was referred to cardiothoracic surgery for resection and excisional biopsy of the mediastinal mass.

## INTRODUCTION

Masses of the mediastinum arise from organs normally located within the mediastinum or traverse through this space [1]. Symptoms of mediastinal masses can include shortness of breath, hoarseness, cough and facial or upper extremity swelling [2]. The mediastinal anterior compartment, bounded anteriorly by the sternum and posteriorly by the pericardium and great vessels, is the most common location of mediastinal masses [3, 4]. The lesions occurring in this region include lymphomas, teratomas, thymic carcinomas and thymomas [3].

Of these, thymic carcinomas and thymomas are the most common neoplasms that arise from the thymus. Approximately 20% of all mediastinal neoplasms are thymomas with a majority of these patients being between the ages of 40 and 60 years of age [5]. In addition to being found incidentally or during workup for chest symptoms, they can also be found during clinical investigation of a paraneoplastic syndrome, which is associated with 40% of thymoma patients [6]. Cytologic configurations within the epithelial cells of the thymus are the key pathologic traits of thymomas. Combining these patterns with perioperative findings of invasion into adjacent structures is the foundation of the Masaoka Classification of Thymic Tumors [7]. Although this classification system serves a degree of prognostic value, the key feature that correlates with survival is tumor invasion [8].

This report examines a rare surgical case of a 22-year-old male patient that underwent en-bloc resection of an enlarged mediastinal mass, which was determined to be a thymoma.

## CASE REPORT

**Figure 1:**
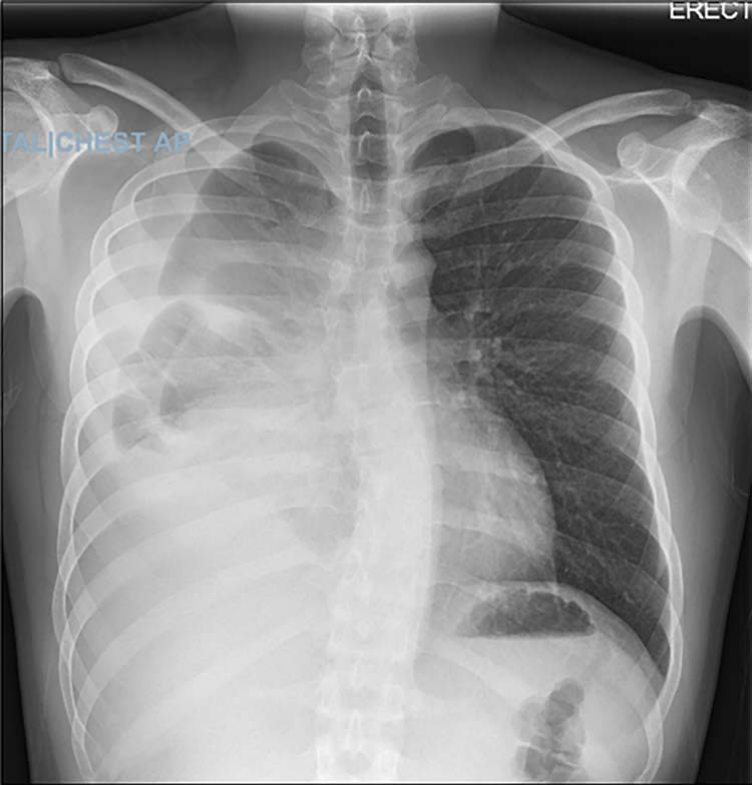
Chest X-ray demonstrating large right pleural effusion.

**Figure 2:**
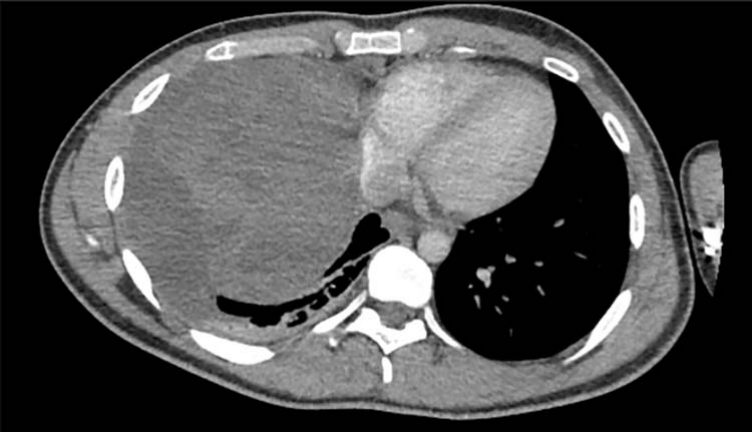
Axial view of CT of thorax with intravenous contrast demonstrating large enhancing and heterogenous mass of the right hemithorax; right-sided pleural effusion is also seen.

A 22-year-old male patient with no significant medical or surgical history and no smoking history presented to the emergency department for evaluation of persistent cough for 3 weeks. The cough was initially associated with chest pain, which then subsided. He denied fever, chills, nasal congestion, shortness of breath and sore throat. A viral panel including COVID-19 was negative and patient was vaccinated against COVID-19. Chest X-ray (Fig. 1) demonstrated a large right pleural effusion with right lower lung consolidation. To further investigate the chest X-ray findings, a computed tomography (CT) of the thorax (Fig. 2) was performed, revealing a 17.2 cm mass occupying the right hemithorax causing a leftward mediastinal shift and narrowing of the right-sided bronchi. To better determine the etiology of the mass, a biopsy was obtained via CT-guided fine needle aspiration. Although analysis of this specimen exhibited mostly necrotic material, it also showed nests of monomorphic epithelial-like cells with many small mature lymphocytes and findings consistent with a thymic neoplasm. Germ cell tumor markers were negative. The beta-hCG, AFP and LDH markers were also negative. However, a more definitive diagnosis was unable to be determined due to the scant amount of collected specimen. Given the clinical, radiographic and pathologic findings, the patient was scheduled for resection of the mass.

**Figure 3:**
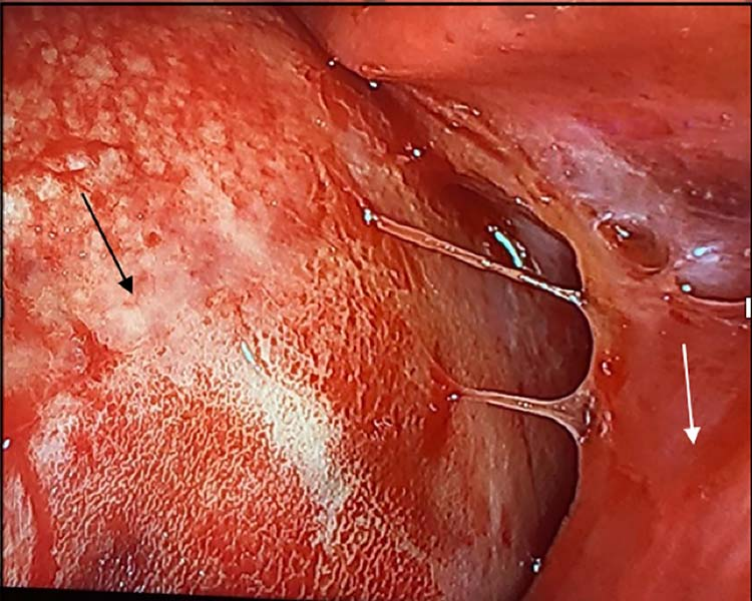
Intraoperative image with patient in left lateral decubitus position demonstrating the mass (black arrow) causing compression of the lung (white arrow).

In the operating room, the patient was placed in the left lateral decubitus position exposing the right side of the chest. Upon entering the pleural cavity, a significant amount of bloody pleural fluid was seen. After fluid aspiration, the mediastinal mass was seen compressing the right lung (Fig. 3). Samples of the tumor were obtained and sent for frozen section analysis, which showed extensive necrosis and desmoplastic reaction, but no evidence of malignancy.

**Figure 4:**
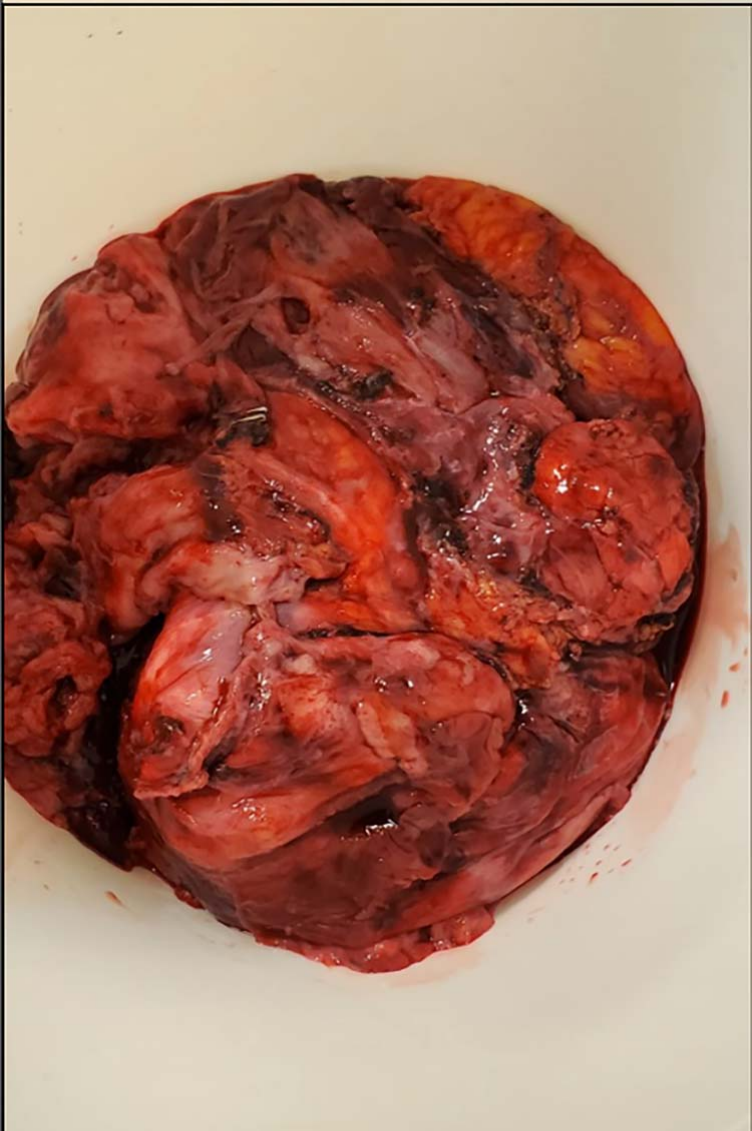
Entirety of the resected mass.

The patient was then turned to the supine position to resect the mediastinal mass, which started with a median sternotomy. The right pleural cavity was entered through the mediastinum, and the mass was mobilized from its adhesions to the chest wall. The lysis of adhesions was continued medially until the hilar structures were visualized. However, mobilization could not be achieved successfully without removing part of the lung that was extremely adherent to the mass. After removing this portion of the lung, the mass also demonstrated adherence to the right lateral aspect of the pericardium. Although multiple attempts were made to preserve the right phrenic nerve, a portion of it, along with part of the pericardium and small portion of normal-appearing thymus, had to be sacrificed due to surrounding inflammation and fibrosis. The mass was then able to be resected and removed, which was then sent for permanent pathologic examination (Fig. 4). before closure, staples were applied in the mediastinum and pericardial surface where the mass was attached to facilitate potential postoperative radiation.

Pathology from the permanent specimen demonstrated a partially encapsulated thymoma along with fibrotic bands that were composed of epithelioid epithelial cell proliferation mixed with many small lymphocytes. Ultimately, the mediastinal mass was determined to be a mixed type B2 and focal B3 thymoma.

The patient was discharged 1 week after surgery. During follow-up visit 1 month following discharge, the patient denied any shortness of breath or any acute symptoms. At this time, the patient was cleared to resume all activities, including heavy lifting. He has been scheduled to receive adjuvant radiation therapy with a repeat CT scan of the thorax in 6 months.

## DISCUSSION

According to the Masaoka Classification of Thymic Tumors, type B2 thymomas demonstrate macroscopic invasion into the thymic or surrounding tissues, and type B3 shows invasion into nearby organs such as the lung or pericardium [9]. Ultimately, this case demonstrates collaboration between various medical specialties, which is essential when addressing the appropriate therapies for thymic tumors [10].

This case warrants discussion of tumor size in thymoma cases, especially since it is considered a critical prognostic factor [11]. Although video-assisted thoracoscopic surgery (VATS) has become more common for surgical resection than a median sternotomy, there still remains ambiguity in terms of limitations and indications for minimally invasive approaches. In addition, there has been controversy regarding whether tumor volume is more valuable than tumor size when deciding the surgical approach [11]. Previous studies have demonstrated a link between tumor diameters exceeding 5 cm and increased risk for thymoma recurrence after VATS [12], as well as tumor volume being more useful than tumor size with regards to choosing between VATS and a median sternotomy [11]. However, there still remains lacking of literature regarding these topics and should be further explored in future studies. Results from these investigations can potentially advance guidelines for surgical treatment of thymomas.

## References

[1] Carter BW, Marom EM, Detterbeck FC. Approaching the patient with an anterior mediastinal mass: a guide for clinicians. J Thorac Oncol 2014;9:S102–9. 25396306 10.1097/JTO.0000000000000294

[2] Henschke CI, Lee IJ, Wu N, et al. CT screening for lung cancer: prevalence and incidence of mediastinal masses. Radiology 2006;239:586–90. 16641357 10.1148/radiol.2392050261

[3] Carter BW, Benveniste MF, Madan R, et al. ITMIG classification of mediastinal compartments and multidisciplinary approach to mediastinal masses. Radiographics 2017;37:413. 28129068 10.1148/rg.2017160095

[4] Aroor AR, Prakasha SR, Seshadri S, S T, Raghuraj U. A study of clinical characteristicsof mediastinal mass. J Clin Diagn Res 2014;8:77–80. 10.7860/JCDR/2014/7622.4013 PMC3972605 24701488

[5] Safieddine N, Liu G, Cuningham K, Ming T, Hwang D, Brade A, et al. Prognostic factors for cure, recurrence and long-term survival after surgical resection of thymoma. J Thorac Oncol 2014;9:1018–22. 24926546 10.1097/JTO.0000000000000215

[6] Morgenthaler TI, Brown LR, Colby TV, Harper CV, Coles DT. Thymoma. May Clinic Proc 1993;68:1110–23. 10.1016/s0025-6196(12)60907-0 8231276

[7] Travis WD et al. World Health Organization International Histological Classification of Tumours. Pathology and Genetics of Tumors of the Lung, Pleura, Thymus and Heart. Lyon: Thymus and Heart IARC Press, 2004.

[8] Chalabreysse L, Roy P, Cordier JF, Loire R, Gamondes JP, Thivolet-Bejui F. Correlation of the WHO schema for the classification of thymic epithelial neoplasms with prognosis: a retrospective study of 90 tumors. Am J Surg Pathol 2002;26:1605–11. 12459627 10.1097/00000478-200212000-00008

[9] Detterbeck FC, Nicholson AG, Kondo K, van Schil P, Moran C. The Masaoka-Koga stage classification for thymic malignancies: clarification and definition of terms. J Thorac Oncol 2011;6:S1710–6. 21847052 10.1097/JTO.0b013e31821e8cff

[10] Girard N, Mornex F, Van Houtte P, Cordier JF, van Schil P. Thymoma: a focus on current therapeutic management. J Thorac Oncol 2009;4:119–26. 19096319 10.1097/JTO.0b013e31818e105c

[11] Miyashita Y, Kanou T, Ishida H, Fukui E, Ose N, Funaki S, et al. Prognostic impact of tumor volume in patients with complete resection of thymoma. Thorac Cancer 2022;13:1021–6. 35166441 10.1111/1759-7714.14353 PMC8977177

[12] Kimura T, Inoue M, Kadota Y, Shiono H, Shintani Y, Nakagiri T, et al. The oncological feasibility and limitations of video-assisted thoracoscopic thymectomy for early-stage thymomas. Eur J Cardiothorac Surg 2013;44:e214–8. 23761417 10.1093/ejcts/ezt305

